# An experimental and theoretical analysis of supercritical carbon dioxide extraction of Cu(II) and Pb(II) ions in the form of dithizone bidentate complexes

**DOI:** 10.55730/1300-0527.3362

**Published:** 2021-12-27

**Authors:** Jeton HALILI, Avni BERISHA

**Affiliations:** Department of Chemistry, FNMS, University of Pristina “Hasan Prishtina”, Pristina, Republic of Kosovo

**Keywords:** Supercritical CO_2_, heavy metals, AAS, DFT

## Abstract

For more than five decades, dithizone has been widely used as an analytical reagent. This ligand forms strongly colored complexes with metal ions and this ability to form complexes can be used for extraction/removal of certain metal ions in addition to analytical determination. In static mode, the supercritical carbon dioxide extraction of copper and lead ions from aqueous solutions after complexation by the dithizone ligand is studied (at two different conditions: a) p = 120 bar, T = 30 °C, and b) p = 72 bar, T = 50 °C). The addition of methanol improved the extraction process by modulating the polarity of the extraction medium. Atomic absorption spectroscopy (AAS) is used to determine the concentration of metal ions before and after extraction. We use density functional theory (DFT) [model chemistry: using m-GGA/M11-L] to better understand the binding energy and geometry of bidentate ligands produced from dithizone and copper(II) or lead(II) ions. Furthermore, the developed complexes’ noncovalent interactions (NCI), bond order analysis, and electron localization function (ELF) provided valuable details about these molecules. To elucidate the bidentate complex extraction mechanism formed between the heavy metal ions and the dithizone ligand, molecular dynamic simulations at periodical boundary conditions were performed using the universal force field to obtain precise molecular descriptions.

## 1. Introduction

Heavy metals are inorganic pollutants with a specific gravity greater than or equal to 5.0 that are extremely toxic and potentially hazardous. Some of these heavy metals are important trace elements, such as Cu, Fe, and Zn, while others, such as Pb, Ni, Cr, and Cd, are relatively toxic [1,2]. Above a certain amount in the human body, however, they become highly toxic. In addition, Pb, Hg, Cd, and As, which are not essential in the human body, are also toxic in trace amounts [3]. Environmental pollution and ecological degradation have become extremely serious due to rapid economic growth, threatening human health and survival. Potentially toxic heavy metal elements contribute significantly to environmental pollution in soil, air, and water [4,5]. Heavy metals are easily absorbed by plants and soils, and thus join the food chain, causing severe harm to human health [1]. Because it can reduce the amount of liquid waste produced and simplify the extraction process, supercritical fluid extraction (SFE) is gaining popularity as a viable alternative to the traditional process [2]. Supercritical fluid extraction is considered a modern technology technique, and there is a growing demand for environmentally friendly metal recovery processes.

The removal of heavy metals from liquid and solid matrices has remained a major problem in recent years. Although several methods are available for this purpose, SFE is considered the most promising technique. Metal ions have been removed from various media with supercritical fluids modified by the use of complexing agents [3].

Because of the charge neutralization required and the weak solute-solvent interaction, direct extraction of metal ions by (SFE) without the addition of a ligand is very inefficient. Metal ions, on the other hand, become extremely soluble in SF-CO_2_ when chelated with organic ligands [4]. Metal ions can be converted to metal chelates by one of two methods. One method is online chelation, in which the ligands are first dissolved in SF-CO_2_ and then flow through the sample matrix. Another approach is in situ chelation, in which the ligands are applied directly to the sample matrix before supercritical fluid extraction (SFE) [5]. According to the literature, both methods of metal ion extraction with SF-CO_2_ have proven successful. When extracting with supercritical CO_2_, many factors must be considered. (1) solubility of the chelating agent, (2) solubility and stability of the metal chelate, (3) density of the supercritical fluid, (4) chemical form of the metal species, 5) addition of modifiers, and (6) sample matrix [6–8].

Several researchers have performed studies on the extraction of heavy metals using SF-CO_2_. Cu extraction from liquid and solid samples using SF-CO_2_ in the presence of Bi’s ligand (trifluoroethyl) dithiocarbamate (FDDC) was demonstrated in 1992 [6]. The SFE of Cu(II) and Zn(II) ions was investigated using static and dynamic extraction techniques with dithizone as a chelating agent [9]. A variety of chelating agents, including dithiocarbamates, β-diketone, reactive organophosphorus compound, macrocyclic compounds, and surfactants, are used for metal complexes in SF-CO_2_.

Molecular mechanics (MM) and density functional theory (DFT) computations have emerged and been embraced as critical tools for studying chemical interactions at many scales and systems. These kinds of computations enable the study of the nature and energetics of molecular interactions, as well as the mechanism of reactions.

The purpose of this work is to investigate the extraction of Pb(II) and Cu(II) ions from aqueous solutions utilizing dithizone as a complexing reagent. Additionally, with the use of advanced theoretical calculations, a molecular knowledge of the interaction, bonding nature, and SF extraction mechanism was gained. To our knowledge, this is the first work to combine experimental and theoretical calculations for supercritical metal extraction utilizing dithizone as a complexing agent.

## 2. Materials and methods

### 2.1. Experimental section

#### 2.1.1. Reagents and instruments

Cu and Pb (Fluka) ICP standard solutions were obtained. Dithizone (MERCK) was used as the chelating agent. Methanol (Sigma Aldrich) was used as the modifier. NaOH (Sigma Aldrich) is used for pH adjustment. Many of the reagents used were analytical grade and of the highest purity. Deionized water was used in all experiments. The metal solutions were prepared from the stock standard solution of Cu(II) and Pb(II). The sample was placed in an extraction cell and an equivalent amount of a chelating agent (dithizone) was added. Carbon dioxide was supplied from a supply tank maintained at a pressure of approximately 53 bar. After the extraction process, samples were digested with HNO_3_ (65%) (Sigma Aldrich) in closed polytetrafluoroethylene (PTFE) tubes using a three-step protocol using microwave (Analytik jena). The concentration of metals such as Cu and Pb obtained during dynamic extraction was determined using an AAS (Analytikjena) equipped with a multi-wave lamp. The air acetylene nebulizer system is used. The flow rate of the nebulizer was 5 ml min^−1^. The wavelengths used are: Cu: 324.8 nm (0.5 nm) and Pb: 217.0 nm (0.5 nm).

**Figure 1:**
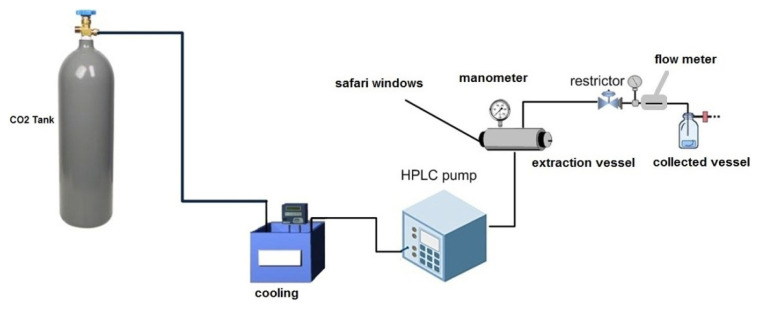
Apparatus for extraction with supercritical CO_2_.

The instrument is calibrated using a 1000 mg/L standard solution of these metals. All metal concentrations are expressed in parts per million (ppm). All experiments were carried out in a supercritical fluid extraction apparatus built by the Department of Chemistry at the University of Prishtina. The supercritical extraction method represented in Figure 1 was used to achieve the study’s objectives. All extractions were performed in a unique stainless steel cylindrical extraction vessel (20 mL) with two glass windows (MAXOS 30 × 15 mm) for phase observation.

#### 2.1.2. Extraction procedure

Static supercritical CO_2_ extraction methods were used. The extraction was carried out under near-critical conditions of liquid CO_2_, temperature (30 °C), pressure (72 bar), and supercritical conditions temperature (50 °C), pressure (120 bar). The dissolved solution was extracted at constant temperature and pressure of CO_2_ for 60 min. After extraction, the solution was removed from the extraction vessel and digested in a microwave oven with HNO_3_ (65%) at 180 °C for 20 min. The solutions were then analyzed using atomic absorption spectrometry (AAS). Extraction efficiencies were calculated based on the amount of metal ions in the water sample before and after extraction.

### 2.2. Computational details

#### 2.2.1. DFT calculations

DFT was performed using the Dmol3 software. Geometry optimization (spin unrestricted) using the double numerical plus polarization basis set (DNP) [10][11] along with the PBE functional within the m-GGA approximation is used [12]. Grimme DFT-D was used to provide dispersion correction effects. [13]. The COSMO method is used to include water as a solvent [14,15]. For the ELF-analysis, a single point geometry calculation (using geometry coordinates generated by the Dmol^3^ software in the previous step) was performed using the Orca software [16] at the density functional theory level with the M06 exchange-correlation functional and the def2-TZVP basis set [17,18]. The van der Waals interactions were accounted for by an atom-pair dispersion correction using the zero-damping scheme (D30) [19]. The adsorption energy, is evaluated using the well-known method [20–22]. The noncovalent interaction (NCI) was calculated using Multiwfn software [23][24]. The NCI surface is plotted using the Visual Molecular Dynamics software [25].

#### 2.2.2. Molecular dynamic

Molecular dynamic simulations were performed at periodical boundary conditions with the universal force field to obtain detailed molecular details to elucidate the extraction process of the bidentate complex formed between the heavy metal ions and the dithizone ligand [26]. The cell with dimensions: 47.2387Å × 47.2387 Å × 75.1343 Å containing an upper layer composed of 296 CO_2_ molecules and a lower layer composed of 2100 water molecules + a Cu(II)-dithizone complex is used in the MD calculations. The MD is performed under NPT ensemble at 323.15 K, p = 0.02 GPa with 1 fs time step and a total simulation time of 500 ps. A Nose thermostat is used for temperature control and a Berendsen barostat was used for pressure control of the system [27][27]. The determination of the self-diffusion coefficient (SDC) [28] is performed by:


(1) $$D=\frac{1}{6}\underset{t\to \infty }{\text{lim}}\frac{d}{dt}\sum_{i=1}^{N\alpha }\langle {\left(r_{i}(t)-r_{i}(0)\right)}^{2}\rangle$$

where the 〈[(r_i_ (t)-r_i_ (0))]^^2^〉 is the mean squared displacement values obtained from MD trajectory.

## 3. Results and discussion

There is a deficit of scientific knowledge on the extraction of heavy metals using SF-CO_2_ and dithizone as chelating agents. Solution pH is an important factor in the extraction of complexes from aqueous samples with supercritical CO_2_. Fischer [29] discovered that all metal-dithizone complexes can exist in the “keto” form, in which the hydrogen atoms of the “phenylimino” groups are substituted by metals (II). In basic solutions or the absence of the dithizone ligand, the metal-dithizone complex can change to the “enol” form (both I and III), which is derived from the “thiol” form of the reagent, which loses two hydrogen atoms (Scheme).

Considering that dithizone contains two dissociated hydrogen atoms, Irving et al. [30] studied dithizone as acid and found the first dissociation constant and the conclusion that the extraction of the metal-dithizone complex is affected by pH.

Due to CO_2_ solubility and carbonic acid formation, the pH of the aqueous phase can decrease (by up to 3) during the extraction process. On the other hand, the metal-dithizone complex is unstable in an acidic environment. Therefore, we used sodium hydroxide to adjust the pH value (pH = 10) in our experiments to increase the pH of the solution. The formation of carbonic acid occurs according to the following reaction.


$${\text{CO}}_{2}+{\text{H}}_{2}\text{O}={\text{H}}_{2}{\text{CO}}_{\text{3}}={\text{H}}^{+}+{{\text{HCO}}^{-}}_{3}$$

Static extraction of Pb(II) and Cu(II) complexes from aqueous solutions was carried out at two different pressures (72 and 120 bar). In these different conditions, the CO_2_ is near critical (72 bar) and at supercritical conditions (120 bar), where a difference in the extraction efficiency is expected.

The extraction percentages of each analyte studied at different pressures are shown in Tables 1 and 2.

In the presence of modifiers, the extraction rate of Pb-dithizone and Cu-dithizone from aqueous solution at 120 bar and 50 °C is greater than 90%, while the extraction rate of these complexes from aqueous solution at near-critical conditions (72 bar and 30 °C) is 95.3% for Pb and 78.80% for Cu. The change in CO_2_ density as a function of pressure explains the lower extraction rate at near-critical conditions.

**Figure 2:**
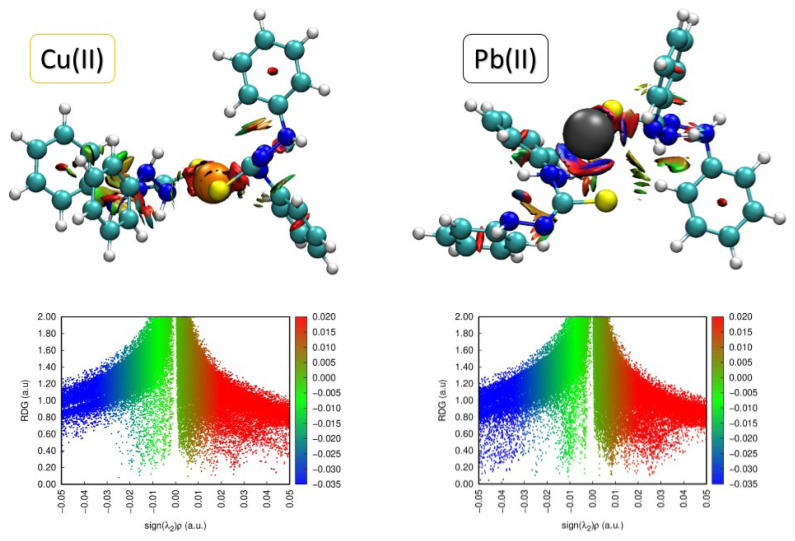
Noncovalent interaction surfaces and the plot of RDG vs. sign(λ)ρ for the van der Waals interactions among the Me(II)/dithizone complexes.

The evaluation of the interaction nature in the formed structures of dithizone complexes is performed via the NCI surface plot and the reduced density gradient (RDG) vs. sign (λ) (Figure 2) [15,31]. The greenish-blueish coloured surface and the spikes with negative sign (λ) values in the 2D NCI plot support that the van der Waals interactions are presented in the formed complex [20,24,32]. It is evident from the NCI plot that these interactions are more strongly present in the Pb(II)/dithizone complex-which is also supported by the interaction energy of this system compared to Cu(II) ions.

**Figure 3:**
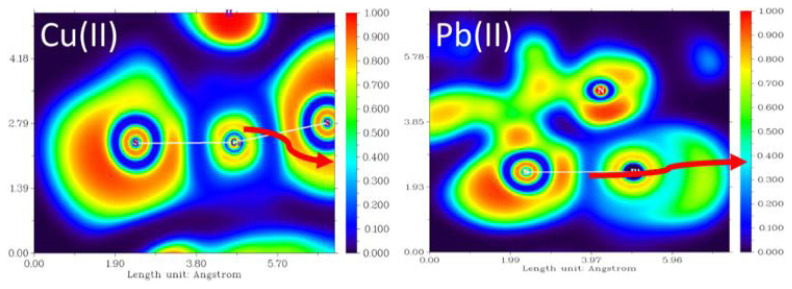
Electron localization function (ELF) analysis of the “bonding” between Me(II) ions and the S-dithizone.

The ‘bonding’ interaction among the metal ions and the dithizone ligand is observable through the ELF analysis (Figure 3), where the low values of ELF indicate the low degree of covalency of these formed bonds [32].

This “binding” is also evident when Mayer’s bonding order analysis is applied [33–35] (Table 3). The Mayer bond order splits the electron density in such a way that the degree of bonding is calculated in a simple way, where a perfectly fulfilled double bond has a value of 2, a triple bond has a value of 3, and so on [35,36]. The bond order values indicate that the interaction of the central metal ions is relatively strong compared to other types of complexes [37].

**Figure 4:**
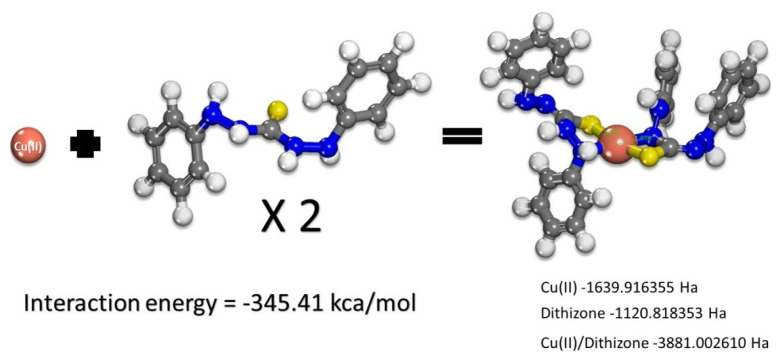
Evaluation of BDE (assessment of interaction strength of Metal/Ligand).

The strength of the interaction (complexation interaction) is calculated using precise quantum chemical methods that account for solvent effects and van der Waals interactions within the system. The interaction energy of the Cu(II)/dithizone ligand is-345.41 kcal/mol, indicating stable complex formation between this ligand/metal pair (Figure 4).

**Figure 5:**
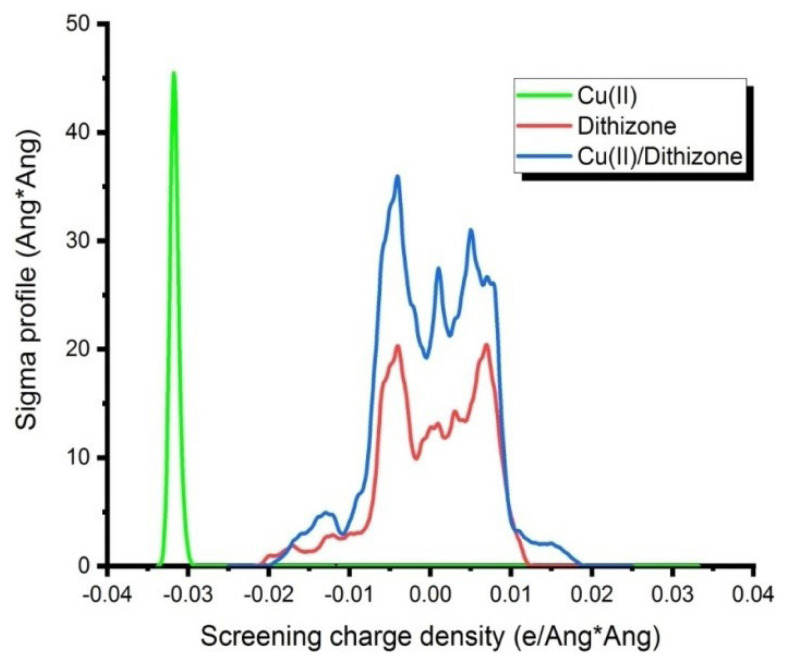
Conductor-like Screening Model–COSMO-Profile.

The sigma profile is indicative of the polarity and hydrogen bond donation or acceptance of the system. The formed complex favours solubility in a polar solvent to some extent and has a nonpolar portion (near zero) that favours CO_2_ extraction (Figure 5).

**Figure 6:**
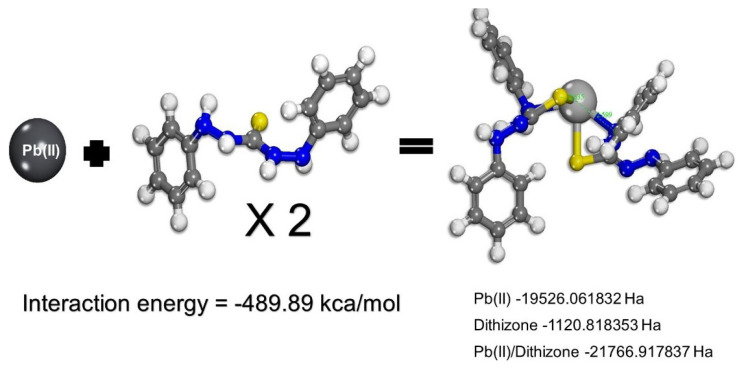
Evaluation of BDE (assessment of interaction strength of Metal/Ligand).

Similarly, the interaction strength between the Pb(II) ions and dithizone is determined for the complex formed using the same methodology. The interaction strength obtained in this case was −489.89 kcal/mol, indicating that the complex formed from lead is more stable than that formed from dithizone (Figure 6).

**Figure 7:**
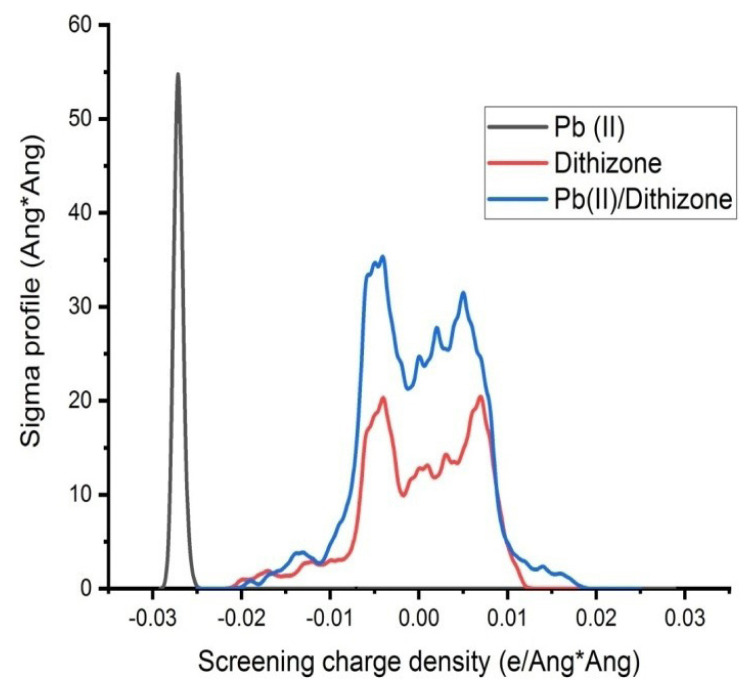
Conductor-like Screening Model–COSMO-Profile.

The same is true for the Pb(II)/dithizone system. The polarity and hydrogen bond donation or acceptance of the system are indicated by the sigma profile, and the complex is polar to some extent, favours solubility from a polar solvent to some extent, and has a nonpolar portion (near zero) that favours CO_2_ extraction (Figure 7).

**Figure 8:**
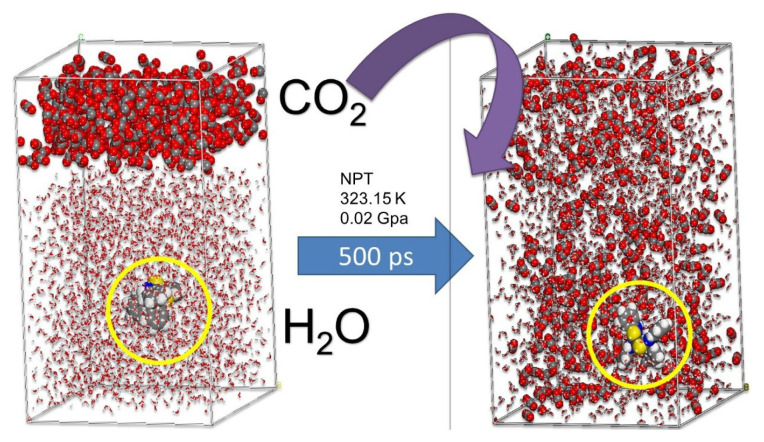
MD simulation of the supercritical carbon dioxide extraction of the Cu(II)-dithizone ligand’s initial and final geometry has been obtained.

A molecular dynamics NPT simulation was performed under the same experimental conditions to obtain information about CO_2_ extraction at the molecular level. The system before a simulation is on the left, and the system after 500 ps simulation time is on the right. As seen in the images, the formed complex is surrounded by CO_2_ molecules, which facilitates the complex’s transition from the aqueous to the CO_2_ phase (Figure 8).

The SDC is an important parameter that defines the molecule’s mobility in the extraction media. This mobility is affected by several factors, including the density and viscosity of the extraction media, polarity, and the interaction of van der Waals with the extraction media [28]. The calculated SDC value is 0.000158913 [cm^2^/s].

## 4. Conclusion

In this study, the heavy metals Pb and Cu were extracted using CO_2_ as fluid under near critical and supercritical conditions. It was shown that the change in pressure and temperature had a significant effect on the extraction efficiency. The positive effect of modifier was also observed in terms of an increase in extraction rate. The extraction efficiency in percentage ranged from Pb(II) (95.3%–99.3%), and for Cu(II) (78.8%–93%) at constant pressure and temperature (30 °C, 72 bar) (50 °C, 120 bar). The values obtained in the extraction near critical conditions are lower than those obtained in the extraction under supercritical conditions. The addition of methanol and NaOH as modifiers significantly affects the growth rate of extraction. As evidenced from DFT calculations there is a strong interaction between Cu(II), Pb(II) ions and dithizone ligand. This interaction is evidenced from ELF and Laplacian bond order calculations. Furthermore, MD provides molecular insights into how the interaction of solvent molecules leads to the uptake of the complexed ions from the extracting solution’s water phase.

## Figures and Tables

**Scheme:**
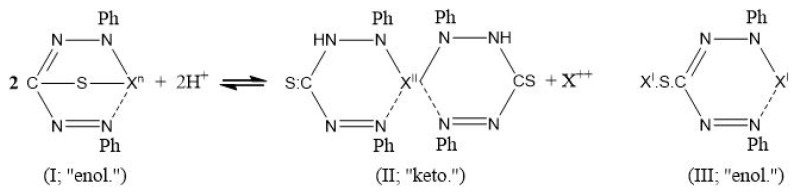
Keto/enol forms of metal-dithizone complexes.

**Table 1:** The mean percent recoveries of Pb by static extraction from SF-CO_2_ using Dithizone as chelating (30 °C, 72 bar) and (50 °C, 120 bar).

Metal	Modifier	72 bar	120 bar
Pb_1_	CH_3_OH	13.7 %	57%
Pb_2_	NaOH+CH_3_OH	95.3 %	99.3%
Pb_3_	NaOH	73.1%	77.2%

**Table 2:** The mean percent recoveries Cu by static extraction from SC-CO_2_ using Dithizon as chelating (30 °C, 72 bar) and (50 °C, 120 bar)

Metal	Modifier	72 bar	120 bar
Cu_1_	CH_3_OH	71.7%	89.1%
Cu_2_	NaOH+CH_3_OH	78.8%	93%
Cu_3_	NaOH	52.7%	81.2%

**Table 3:** Mayer bond order for selected bonding atoms in the dithizone complexes.

Metal ion	Bonding atoms	Mayer bond order
**Pb(II)**	Pb(1)-S(2)	0.790
	Pb(1)-S(33)	0.618
	Pb(1)-N(3)	0.250
**Cu(II)**	Cu(1)-S(2)	0.820
	Cu(1)-S(3)	0.808
	Cu(1)-N(3)	0.491
